# Chelerythrine induces apoptosis via ROS‐mediated endoplasmic reticulum stress and STAT3 pathways in human renal cell carcinoma

**DOI:** 10.1111/jcmm.14295

**Published:** 2019-09-30

**Authors:** Hongchao He, Ran Zhuo, Jun Dai, Xiaojing Wang, Xin Huang, Haofei Wang, Danfeng Xu

**Affiliations:** ^1^ Ruijin Hospital, School of Medicine Shanghai Jiaotong University Shanghai China

**Keywords:** apoptosis, Chelerythrine, endoplasmic reticulum stress, ROS, STAT3

## Abstract

Renal cell carcinoma (RCC) is a heterogeneous histological disease and it is one of the most common kidney cancer. The treatment of RCC has been improved for the past few years, but its mortality still remains high. Chelerythrine (CHE) is a natural benzo[c]phenanthridine alkaloid and a widely used broad‐range protein kinase C inhibitor which has anti‐cancer effect on various types of human cancer cells. However, its effect on RCC has not been fully elucidated. In this study, we evaluated the effect and mechanism of CHE on RCC cells. Our study showed that CHE induced colony formation inhibition and G2/M cell cycle arrest in a dose‐dependent manner in RCC cells. In addition, CHE increased cellular ROS level, leading to endoplasmic reticulum (ER) stress, inactivating STAT3 activities and inducing apoptosis in RCC cells which were suppressed by NAC, a special ROS inhibitor. We further found that both knockdown of ATF4 protein and overexpression of STAT3 protein could reduce CHE‐induced apoptosis in Caki cells. These results demonstrated that the apoptosis induced by CHE was mediated by ROS‐caused ER stress and STAT3 inactivation. Collectively, our studies provided support for CHE as a potential new therapeutic agent for the management of RCC.

## 1 INTRODUCTION

Renal cell carcinoma (RCC) is one of the most common cancers in the urinary system, accounting for 2%‐4% of all malignant adult diseases worldwide, with a mortality rate of over 40%.^1^, ^2^ RCC is always asymptomatic in its early stages, therefore, the disease often develops into advanced stages at the time of diagnosis and approximately 25%‐30% of patients have metastatic RCC (mRCC) at diagnosis.^3^ There are currently several methods for treating RCC, such as radical surveillance, targeted therapy, conventional chemotherapy, and/or immunotherapy.^3^ Unfortunately, about 40% of patients are resistant to conventional chemotherapy and radiation therapy. These patients may experience systemic recurrence, with the consequent high toxicity and low response treatment failure. The data show a 2‐5 year survival rate of RCC is less than 20%.^4^, ^5^ Therefore, there is an urgent need to develop an effective and tolerable therapy for RCC.

In recent years, Chinese medicine has been widely explored by clinical and medical researchers which offer additional advantages of being less expensive than conventional drugs.^6^, ^7^ Approximately 60% of clinically used anti‐cancer drugs are natural products or derivatives.^8^ Chelerythrine (CHE) is a natural benzo[c]phenanthridine alkaloid extracted from plant species, such as Chelidonium majus, Macleaya cordata, and Sanguinaria canadensis et al.^9^, ^10^ CHE has a wide range of biological activities and plays an important role in anti‐diabetes,^11^ anti‐cancer,^12^ anti‐fungus,^13^ and the lipopolysaccharide‐induced endotoxic shock^14^ etc. The anti‐cancer effects of CHE have been studied both in vitro and in vivo. Regulation of Bcl‐2 family protein expression and activation of the mitochondrial pathway were reported to be associated with CHE induced hepatoma cell apoptosis.^15^ in addition, CHE can inhibit proliferation and promote apoptosis in prostate cancer,^16^ triple‐negative breast cancer,^17^ non‐small cell lung cancer^12^ (NSCLC) etc.

Here in the present study, we aimed to investigate the effect of CHE on the cultured human RCC. The results showed that CHE inhibited the growth of human RCC, induced G2/M cell cycle arrest and apoptosis. It also demonstrated that CHE induced apoptosis in human RCC through ROS mediated endoplasmic reticulum (ER) stress pathway. Moreover, we discovered for the first time that CHE induced apoptosis in human RCC through the inactivation of signal transducer and activator of transcription‐3 (STAT3) signalling pathway. In summary, our findings indicated that CHE might be a therapeutic candidate in the treatment of RCC and a novel mechanism of CHE in anti‐cancer activities was reported in this study.

## 2 METHODS

### 2.1 Cell culture and reagents

Human renal cancer cell lines (Caki and 786‐O), human normal hepatocytes cell lines (LO2) were purchased from the Institute of Biochemistry and Cell Biology, Chinese Academy of Sciences (Shanghai, China). All cells were cultured in McCoy's medium or RPMI 1640 media (Gibco, Eggenstein, Germany) supplemented with 10% heat‐inactivated foetal bovine serum (FBS; Hyclone, Logan, UT), 100 units/mL penicillin, and 100 µg/mL streptomycin. CHE was obtained from Aladdin (Shanghai, China). Antibodies including anti‐Bax, anti‐Bcl‐2, anti‐Cdc2, anti‐Cyclin B1, anti‐GAPDH, anti‐MDM‐2, donkey anti‐rabbit IgG‐HRP and goat anti‐mouse IgG‐HRP horseradish peroxidase were purchased from Santa Cruz Biotechnology (Santa Cruz, CA). Antibodies including anti‐Cle‐PARP, anti‐p‐eIF2α, anti‐eIF2α, anti‐ATF4, anti‐p‐STAT3 and anti‐STAT3 were purchased from Cell Signaling Technology (Danvers, MA). dimethylsulfoxide (DMSO), methyl thiazolyl tetrazolium (MTT), N‐acetyl cysteine (NAC), Trolox, Catechin hydrate (CTH), Vitamin E (Vita‐E) and Butylated hydroxyanisole (BHA) were obtained from Sigma‐Aldrich (St. Louis, MO).

### 2.2 Cell viability assay

To measure viability of cells, we plated 6 × 10^3^ cells per well of a 96‐well plate. Cells were attached overnight in complete growth media and were treated with CHE (dissolved in DMSO; diluted in RPMI medium) for 24 h, after which were subjected to the MTT assay.

### 2.3 Colony formation assay

Caki and 786‐O cells were seeded at 600 cells per well in six‐well plates and treated with 2, 4 or 8 µmol/L CHE for 10 hours. Cells were allowed to grow for 8 or 9 days and stained with crystal violet solution to assess colony growth.

### 2.4 Cell cycle and apoptosis analysis

For cell cycle analysis, cells were treated with CHE (6, 9 or 12 μmol/L) for 20 hours. The cells were then stained with PI (BD Biosciences, San Jose, CA) at a final concentration of 0.05 mg/mL and incubated at 4°C for 10 min in the dark. Cell cycle analysis was performed in Accuri C6 plus flow cytometer (BD Biosciences, CA).

For apoptosis determination, cells were treated with CHE (6, 9 or 12 μmol/L) for 24 hours. Cells were then harvested and washed with PBS, resuspended in binding buffer containing Annexin V and propidium iodide (PI) (BD Biosciences, San Jose, CA). Cell apoptosis analysis was performed in Accuri C6 plus flow cytometer (BD Biosciences, CA).

### 2.5 Determination of intracellular ROS

Intracellular ROS levels were measured through flow cytometry using DCFH‐DA. In short, 5 × 10^5^ cells were seeded in six‐well plates, attached overnight, and then treated with CHE (6, 9 or 12 μmol/L) for 3 hours. NAC pretreatment for 1 hours if required. Cells were stained with 10 μmol/L DCFH‐DA at 37°C under dyeing 30 minutes in the dark. Analysis of DCF fluorescence in the presence of ROS using Accuri C6 plus flow cytometer.

### 2.6 Western blot analysis

Lysates from cells were prepared to determine protein levels using the Bradford assay (Bio‐Rad, Hercules, CA). Proteins were separated by 10% SDS‐PAGE and transferred to poly‐vinylidene difluoride transfer membranes. The blots were blocked with freshly prepared 5% nonfat milk in TBST for 2 hours at room temperature. Then the blots were incubated with specific primary antibodies overnight at 4°C. HRP‐conjugated secondary antibodies and ECL substrate (Bio‐Rad) were used for detection.

### 2.7 Electron microscopy

Caki cells were seeded in 60 mm plates and then were treated with 12 μmol/L CHE in the presence or absence of NAC (5 mmol/L). The collected cells were fixed in phosphate buffer (pH 7.4) including 2.5% glutaraldehyde for 12 hours at 4°C. The cells were post‐fixed in 1% OsO4 for 60 min at room temperature, stained with 1% uranyl acetate, dehydrated by graded acetone solutions and embedded in Epon. Areas containing cells were block‐mounted and cut into 70 nm sections and examined with the electron microscope (H‐7500, Hitachi, Ibaraki, Japan).

### 2.8 Cell transfections

To knockdown ATF4 expression, Caki cells were seeded in six‐well plates at a density of 6 × 10^4^ and cultured for 24 hours. siRNA against ATF4 or non‐targeting control were transfected in a final concentration of 50 pmol mL^−1^ using lipofectamine 3000 reagent (Invitrogen, CA). After 6‐8 hours, the medium was replaced with fresh medium and cells were cultured for 36 hours. Then, cells were treated with 12 μmol/L CHE for 3 hours and used for subsequent experiments. siRNA oligonucleotides purchased from GenePharma (Shanghai, China). ATF4 siRNA sequences: (sence 5′‐GCCUAGGUCUCUUAGAUGATT‐3′; antisense, 5′‐UCAUCUAAGAGACCUAGGCTT‐3′).

To express STAT3, the recombinant plasmid vector coding STAT3 protein was purchased from Addgene (Plasmid #71450, Addgene, Cambridge, MA). According to the manufacturer's protocol, human renal cancer cell line (Caki) was transfected by STAT3 plasmid through Lipofectamine 3000 reagent (Invitrogen, Carlsbad, CA). After 36‐48 hours of transfection, the protein of STAT3 expression was detected by Western blotting analysis.

### 2.9 Statistical analysis

All experiments were repeated at least three times. Statistical analyses were performed only when a minimum of n = 3 independent samples were acquired. All data were expressed as mean ± SEM. Statistical analysis was performed with GraphPad Prism 6.0 software (GraphPad, San Diego, CA).

## 3 RESULTS

### 3.1 CHE reduces cell viability in human renal cancer cells

**Figure 1:**
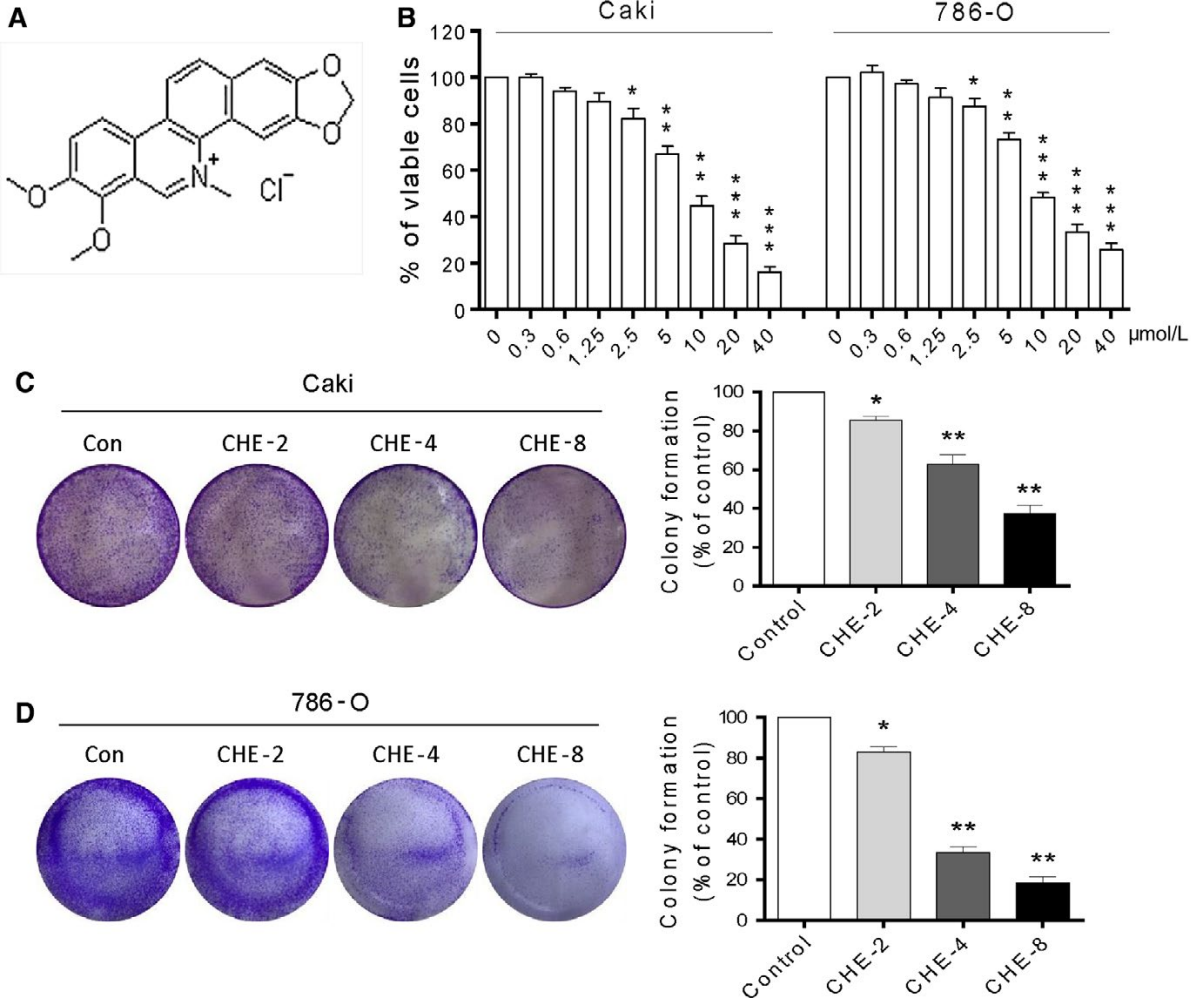
Chelerythrine (CHE) reduces cell viability in human renal cancer cells. (A) The chemical structure of CHE. (B) Cell viability was analysed using methyl thiazolyl tetrazolium cell proliferation assay kits. The proliferation of Caki and 786‐O cells treated with CHE was significantly decreased in a dose‐dependent manner for 24 h. (C–D) Effect of varying CHE concentrations on renal cancer cell colony formation. Cells were incubated with CHE for 10 h and allowed to grow for 8‐9 days. Colonies were stained by crystal violet dye. The colony formation ability of each group was shown in bar chart. All images shown here are representative of three independent experiments with similar results. Data are shown as mean ± SEM (n = 3). *P < 0.05, **P < 0.01 and ***P < 0.001 compared with the dimethylsulfoxide group

The structure of CHE is shown in Figure 1A. CHE was reported to reveal inhibitory activity in various human cancers. To investigate whether CHE exhibits similar inhibitory role in human renal cancer cells, we first assessed the viability of human renal cancer cells after exposure to CHE. Caki and 786‐O cells were challenged with increasing concentrations of CHE and the number of viable cells were measured by the MTT assay. As shown in Figure 1B, after treatment with CHE for 24 hours, the viability of Caki cells and 786‐O cells decreased significantly in a dose‐dependent manner. The IC_50_ values were 7.62 and 8.45 μmol/L for 24 hours. We also tested the cytotoxic effects of CHE on cultured human normal hepatocytes cells (LO2). The viability of normal LO2 cells was affected only minimally at the highest concentration (10 μmol/L) tested (Figure S1). Then we used the colony formation assay to explore whether Caki and 786‐O cells could form colonies after the CHE treatment. The data showed that CHE prevented colony formation in a dose dependent manner (Figure 1C,D). Taken together, our results showed that CHE could selectively kill human renal cancer cells but not normal cells.

### 3.2 CHE induces cell apoptosis in human renal cancer cells

**Figure 2:**
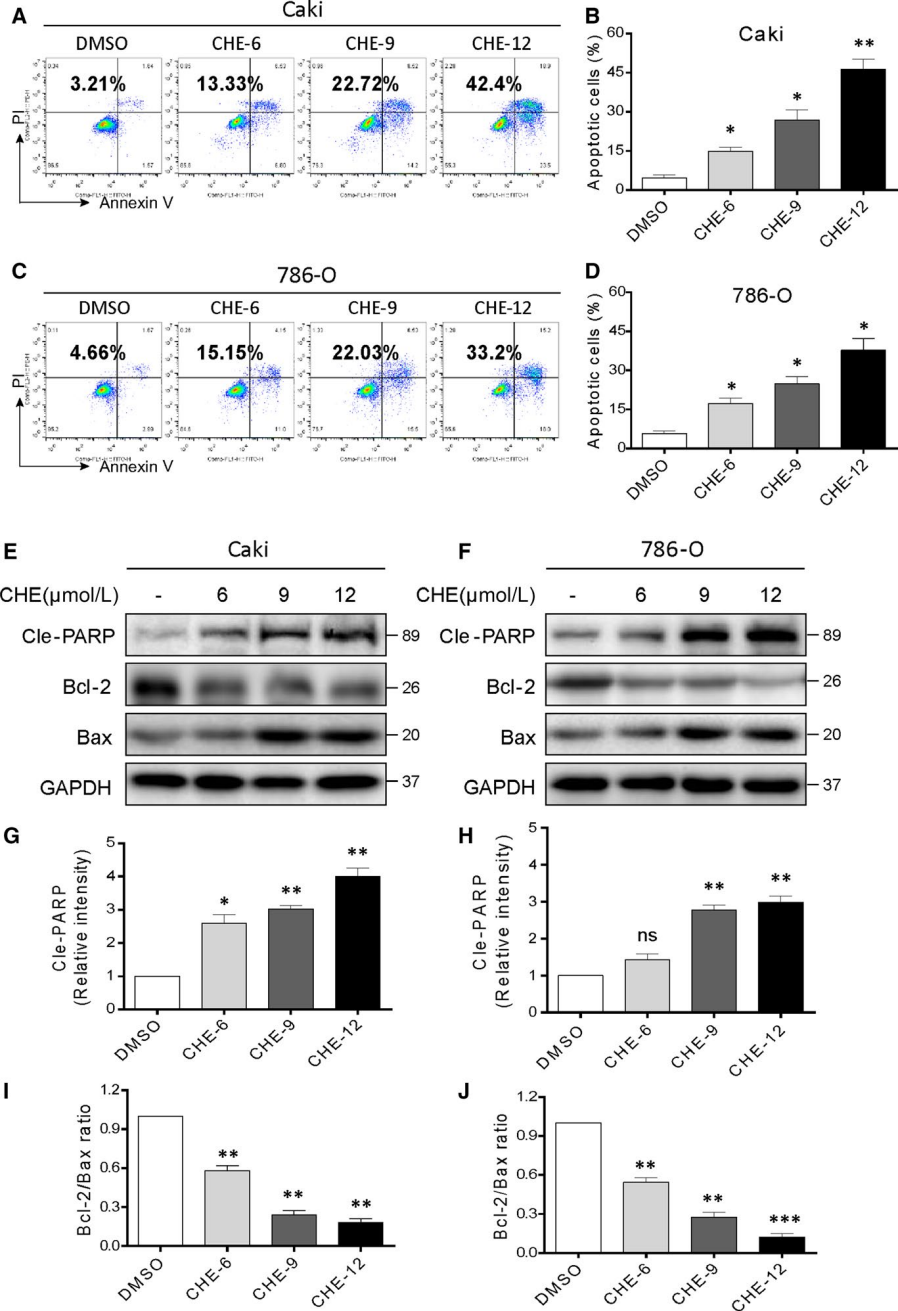
Chelerythrine (CHE) induces apoptosis in human renal cancer cells. (A, C) Caki and 786‐O cells were exposed to CHE at the indicated concentrations for 24 h. Percentage of cell apoptosis was determined by Annexin‐V/PI staining and flow cytometry. Similar results were obtained in three independent experiments. (B, D) The percentage of apoptotic cells in the treatment groups was quantified. (E) Expression of apoptosis‐related proteins Cle‐PARP, Bcl‐2 and Bax were determined by western blot after treatment with CHE (6, 9 or 12 μmol/L) for 20 h in Caki renal cancer cells. GAPDH was used as internal control. (F, G) Quantification of data presented in panel E. (H) Expression of apoptosis‐related proteins Cle‐PARP, Bcl‐2 and Bax were determined by western blot after treatment with CHE for 20 h in 786‐O cells. GAPDH was used as internal control. (I, J) Quantification of data presented in panel H. All data here are expressed as means ± SD of triplicates. All images shown here are representative of three independent experiments with similar results. Data are shown as mean ± SEM (n = 3). *P < 0.05, **P < 0.01 and ***P < 0.001 compared with the dimethylsulfoxide group

Reduced viability in human RCC after CHE exposure prompted us to determine whether CHE induced apoptosis. We investigated the pro‐apoptosis effects of CHE using Annexin VFIT + PI staining by flow cytometry analysis. Renal cancer cell lines showed dose‐dependent apoptotic cell death for 24 hours after CHE treatment (Figure 2A‐D). To further confirm these findings, we detected the apoptosis‐related proteins in renal cancer cells. Our results showed that CHE increased the expression of cleavage poly ADP‐ribose polymerase (Cle‐PARP) dose‐dependently in two renal cancer cell lines (Figure 2E,F,H,I). In addition, CHE treatment decreased the protein level of B‐cell lymphoma‐2 (Bcl‐2) and increased the Bcl‐2 associated X protein (Bax) level in two renal cancer cell lines (Figure 2E,H). The decreased Bcl‐2: Bax ratio indicated induction of apoptosis in cells (Figure 2G,J).

### 3.3 CHE causes cell cycle arrest in human renal cancer cells

We analysed the effect of CHE treatment on cell cycle distribution in human renal cancer cells. We treated the cells with increasing concentrations of CHE and then evaluated cell cycle phase distribution by flow cytometry. CHE induced G2/M cell cycle arrest in Caki and 786‐O cells (Figure S2A‐D). We observed the greatest accumulation of cells in the G2/M phase upon 12 µmol/L CHE exposure in Caki as well as 786‐O cells. Western blotting analysis also showed that CHE dose‐dependently reduced the expression of cell cycle related proteins such as Cyclin‐dependent kinase 1 (CDC2), Cyclin B1 and Mouse double minute 2 homolog (MDM‐2) in human renal cancer cells (Figure S2E‐H). Together, these results suggested that CHE treatment reduced viability of renal cancer cells involving G2/M phase arrest and apoptotic cell death.

### 3.4 CHE increases ROS levels in human renal cancer cells

**Figure 3:**
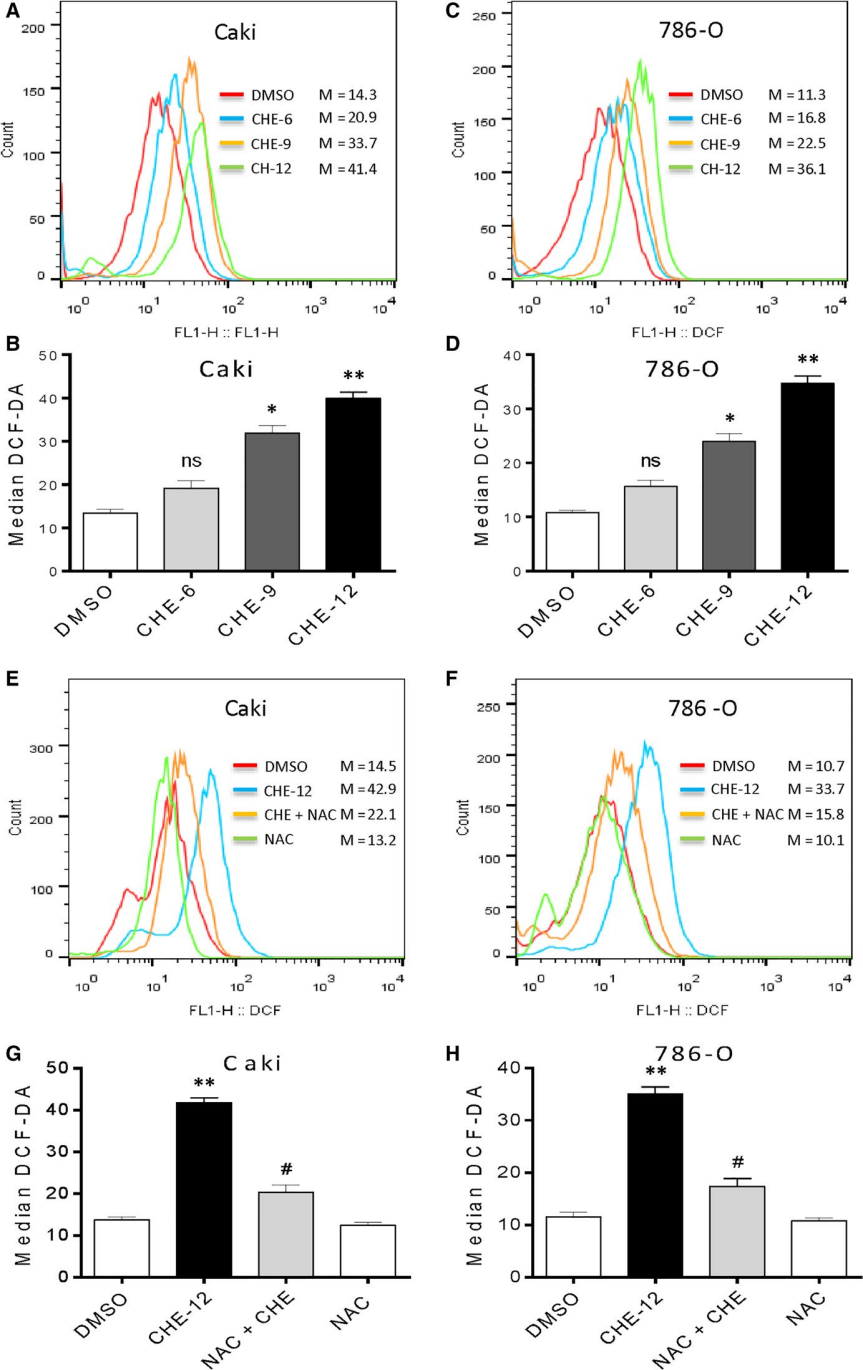
Chelerythrine (CHE) induces ROS accumulation in human renal cancer cells. (A, C) Intracellular ROS generation dose‐dependently induced by CHE was measured in Caki and 786‐O cells by staining with DCFH‐DA (10 μmol/L) and flow cytometry analysis. Caki and 786‐O cells were treated with CHE at the indicated concentrations for 3 h. Then, Intracellular ROS generation was measured by flow cytometry. (B, D) Quantification of data presented in panel A and C. (E, G) Effect of NAC pretreatment of 1 h on ROS levels. Relative fluorescence intensity was assayed by flow cytometer. (F, H) Quantification of data presented in panel E and G. All data here are expressed as means ± SD of triplicates. All images shown here are representative of three independent experiments with similar results. Data are shown as mean ± SEM (n = 3). *P < 0.05, **P < 0.01 compared with the dimethylsulfoxide group; ^#^ *P* < 0.05 compared with the CHE‐12 group

Previous studies have been shown that CHE may increase ROS level in prostate cancer cells and non‐small cell lung cancer cells.^12^, ^16^ Hence, we investigated the effects of CHE on treatment renal cancer cells to produce ROS. We firstly performed flow cytometry analysis using DCFH‐DA fluorescent dyes. As shown in Figure 3A‐D, 6‐12 µmol/L CHE treatment in Caki and 786‐O cancer cells, the results showed that CHE could dose‐dependently increase the ROS level in renal cancer cells. However, pretreatment with NAC (the specific ROS inhibitor) for 1 hour significantly suppressed the ROS levels caused by CHE (Figure 3E‐H). We also used other 4 ROS scavengers (BHA, Vita‐E, Trolox and CTH) to narrow ROS species. Interestingly, all of these data did not reverse the ROS generation induced by CHE (Figure S3) and clearly demonstrated that CHE increased ROS levels in renal cancer cells. Besides, only NAC inhibited CHE‐induced ROS, while other lipid ROS inhibitors or lipid peroxide quenchers did not have this effect.

### 3.5 CHE induces ROS‐mediated apoptosis and G2/M cell cycle arrest in human renal cancer cells

**Figure 4:**
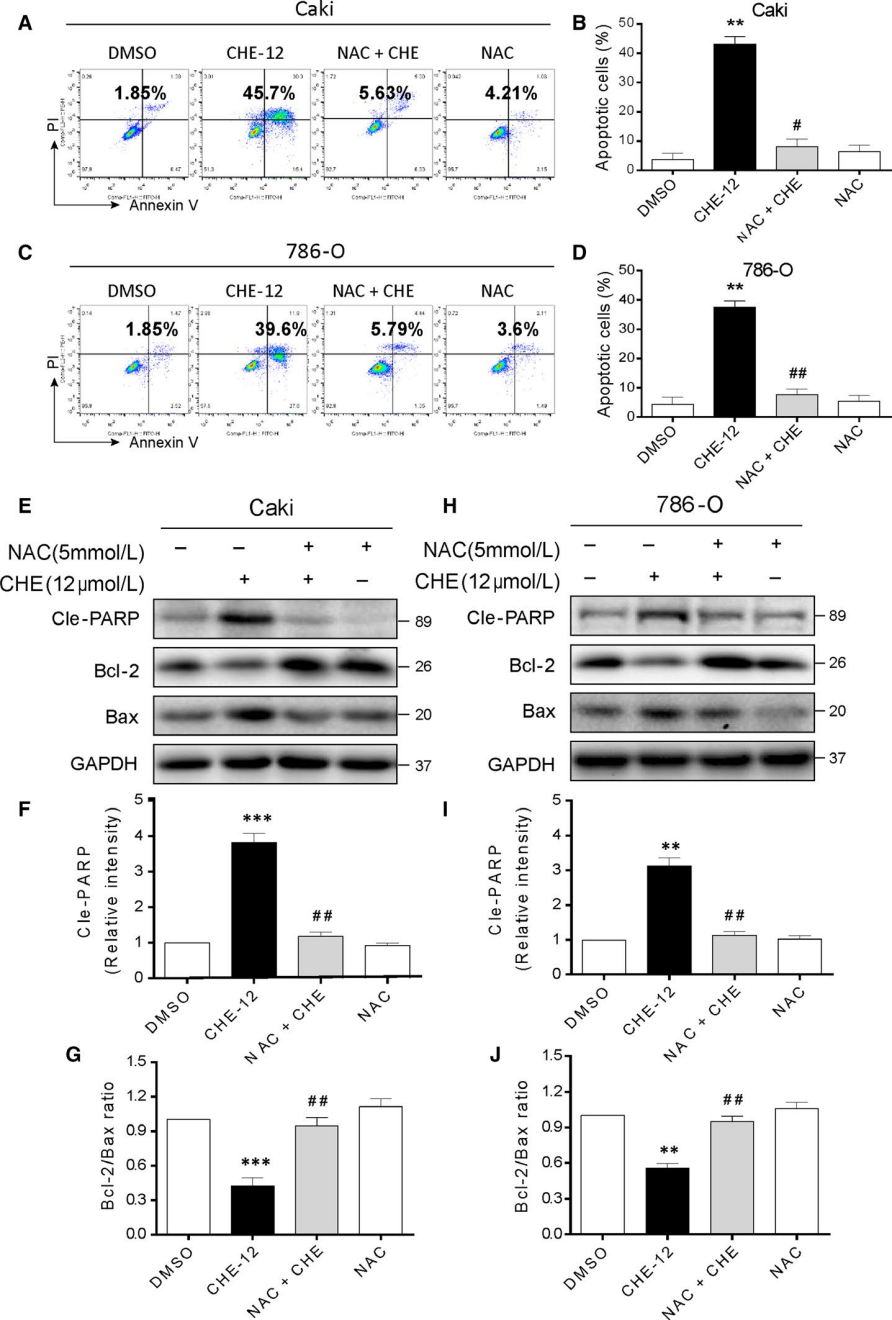
Chelerythrine (CHE) induces ROS‐dependent apoptosis in human renal cancer cells. (A, C) Caki and 786‐O cells were pre‐incubated with or without 5 mmol/L NAC for 1 h before exposure to CHE at the indicated concentrations for 24 h. Percentage of cell apoptosis was determined by Annexin‐V/PI staining and flow cytometry. Similar results were obtained in three independent experiments. (B, D) The percentage of apoptotic cells in the treatment groups was quantified. (E) Expression of apoptosis‐related proteins Cle‐PARP, Bcl‐2 and Bax were determined by western blot after treatment with CHE (12 μmol/L) or CHE (12 μmol/L) + NAC (5 mmol/L) pretreated or NAC (5 mmol/L) for 20 h in Caki renal cancer cells. GAPDH was used as internal control. (F, G) Quantification of data presented in panel E. (H) Expression of apoptosis‐related proteins Cle‐PARP, Bcl‐2 and Bax were determined by western blot after treatment with CHE (12 μmol/L) or CHE (12 μmol/L)+NAC (5 mmol/L) pretreated or NAC (5 mmol/L) for 20 h in 786‐O renal cancer cells. GAPDH was used as internal control. (I, J) Quantification of data presented in panel H. All images shown here are representative of three independent experiments with similar results. Data are shown as mean ± SEM (n = 3). **P < 0.01 and ***P < 0.001 compared with the dimethylsulfoxide group; ^#^ *P* < 0.05, ^##^ *P* < 0.01 compared with the CHE‐12 group

Next, we designed experiments to identify the important role of ROS in mediating CHE's anti‐cancer effects in human renal cancer cells. Pretreatment with 10 mmol/L NAC for 1 hour and then treated with 12 µmol/L CHE for 24 hour in Caki and 786‐O cells. As shown in Figure 4A‐D, pretreatment with NAC in renal cancer cells almost completely reversed cell apoptosis caused by CHE. However, the other 4 ROS scavengers did not have this effect (Figure S4). The results showed that thiol‐containing antioxidants (NAC) suppressed CHE‐induced ROS and apoptosis. Besides, the ROS effects were further validated through western blot analysis. The changes induced by CHE in Cle‐PARP, Bax and Bcl‐2 were all reversed by NAC pre‐treatment (Figure 4E‐J). We next explored whether the cell cycle arrest was mediated by CHE‐induced ROS accumulation in renal cancer cells. The results showed that co‐treatment with NAC totally reversed CHE‐induced G2/M cell cycle arrest in human renal cancer cells (Figure S5A‐D). Western blotting analysis also indicated that pretreatment with NAC notably prevented the down‐regulation of cell cycle‐related proteins, such as CDC2, MDM‐2 and Cyclin B1 in human renal cancer cells (Figure S5E‐H). All these findings fully demonstrated that CHE caused ROS‐dependent G2/M cell cycle arrest and apoptosis in human renal cancer cells.

### 3.6 CHE caused ROS‐dependent ER stress activation in human renal cancer cells

**Figure 5:**
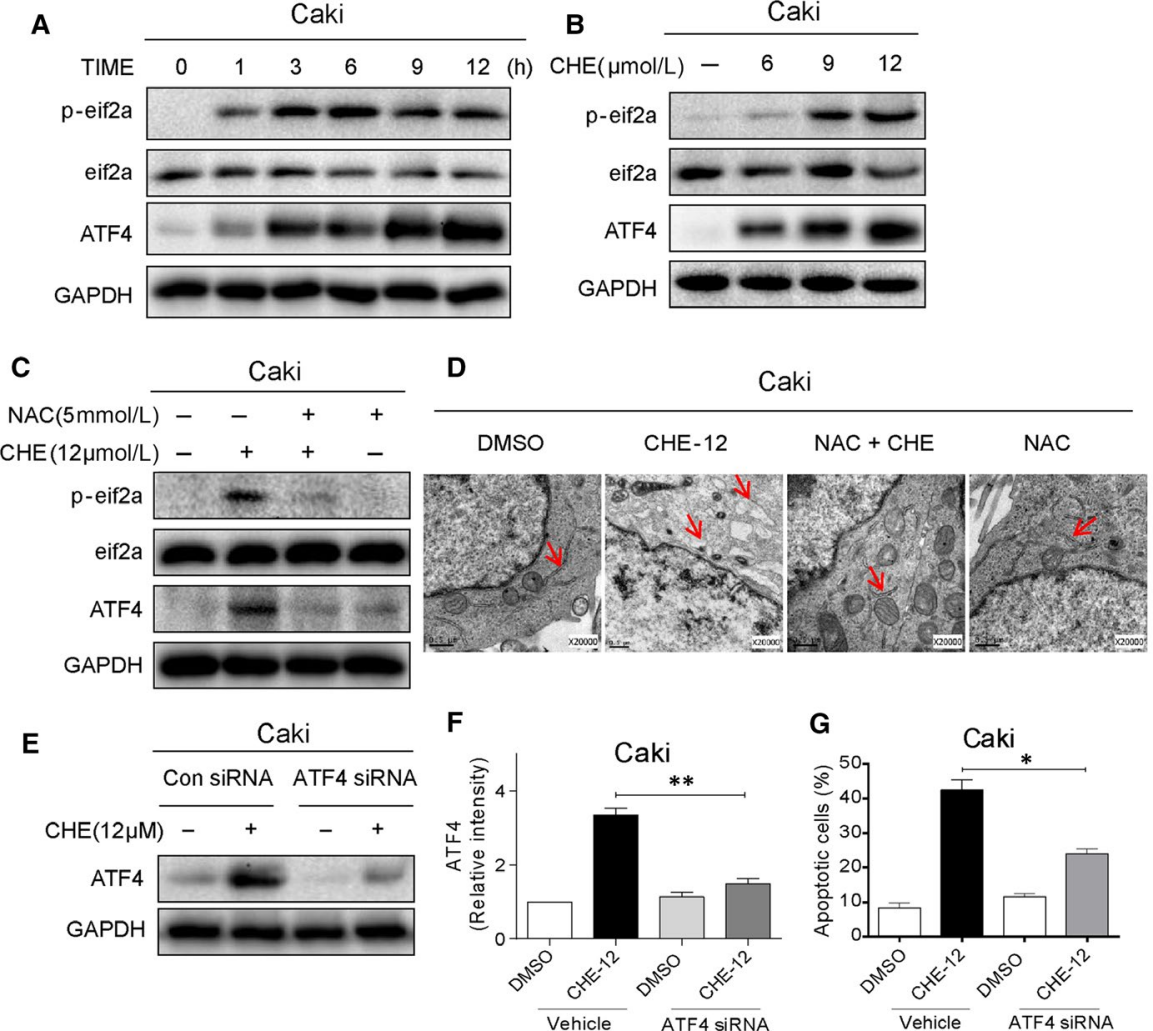
Chelerythrine (CHE) induces apoptosis in renal cancer cells by ROS‐dependent endoplasmic reticulum (ER) stress pathway. (A) Expression of ER‐stress pathway in renal cancer cells as assessed by protein induction of phosphorylated eIF2α and ATF4. Cells were exposed to 12 μmol/L CHE for different time periods. eIF2α and GAPDH served as controls. (B) Western blot analysis of ER‐stress pathway associated proteins in cells exposed to various concentrations of CHE for 3 h (ATF‐4 and p‐EIF2α). (C) Effect of NAC pretreatment on CHE‐induced ER stress pathway proteins. NAC was used at 5 mmol/L for 1 h before exposure to CHE. (D) Electron microscopy images of Caki cells exposed to CHE [×20 000 shown]. Cells were exposed to 12 μmol/L CHE for 8 h. (E) Western blot analysis of ATF4 protein following siRNA transfection in Caki cells [Con siRNA = negative control siRNA transfected cells treated with vehicle, Con siRNA + CHE = negative control siRNA transfected cells treated with CHE, ATF4 siRNA = ATF4 siRNA transfected cells treated with vehicle, ATF4 siRNA + CHE = ATF4 siRNA transfected cells treated with CHE]. (F, G) Effect of ATF4 knockdown on CHE‐induced apoptosis as assessed by Annexin V/PI staining. Caki cells were transfected with ATF4 siRNA for 24 h and then exposed to 12 μmol/L CHE. All images shown here are representative of three independent experiments with similar results. Data are shown as mean ± SEM (n = 3). *P < 0.05, **P < 0.01

Previous studies have proved that CHE causes prostate cancer cell apoptosis mainly related to the activation of ER stress signalling pathway.^16^ Besides, it has been report that oxidative stress modulating drugs activate the ER stress‐related apoptosis.^18^ Thus, we speculated that ER stress contributed to renal cancer cells apoptosis by CHE treatment. Activating transcription factor 4 (ATF4) is a key transcription factor in the ER stress pathway. Next, we determined the protein expression which is associated with ER stress, such as ATF4 and p‐eIF2α in CHE‐treated Caki cells. Western blot results indicated that CHE (12 μmol/L) could time dependently activate ER stress (Figure 5A). We also found that CHE could dose‐dependent increase the p‐eIF2α and ATF4 protein expression in Caki cells (Figure 5B). As shown in Figure 5C, pre‐treatment with NAC could completely reverse the CHE‐induced changes of ER stress‐related proteins. To further test effects of CHE, we directly observed the morphology of ER in Caki cells through electronic microscopy and DMSO‐treated Caki cells (×20000 amplification) showed the normal appearance of smooth ER (arrow). Treatment with CHE (12 μmol/L) for 8 hour made the ER swelling (arrow) in Caki cells, which suggested the misfolded protein was accumulated in ER (Figure 5D). However, pretreatment with NAC (5 mmol/L) could reverse this morphological alteration in Caki cells, while treatment with NAC (5 mmol/L) alone had no effect on the ER morphology (Figure 5D). To confirm the upstream role of ER‐stress pathway during the renal cancer apoptosis, we assessed the effect of CHE after altering ATF4 levels in Caki cells. The ATF4 expression markedly reduced after knockdown of ATF4 by siRNA in Caki cells (Figure 5E,F). We assumed that if ATF4 (ER stress) was involved in CHE‐mediated Caki cell death, the cell apoptosis induced by CHE would be expected to decrease after knocking it down. As shown in Figure 5G, the data clearly demonstrated our hypothesis, ATF4 knockdown reduced apoptotic cell death by CHE.

### 3.7 CHE inactivates STAT3 activity, which contributes to CHE lethality in human renal cancer cells

**Figure 6:**
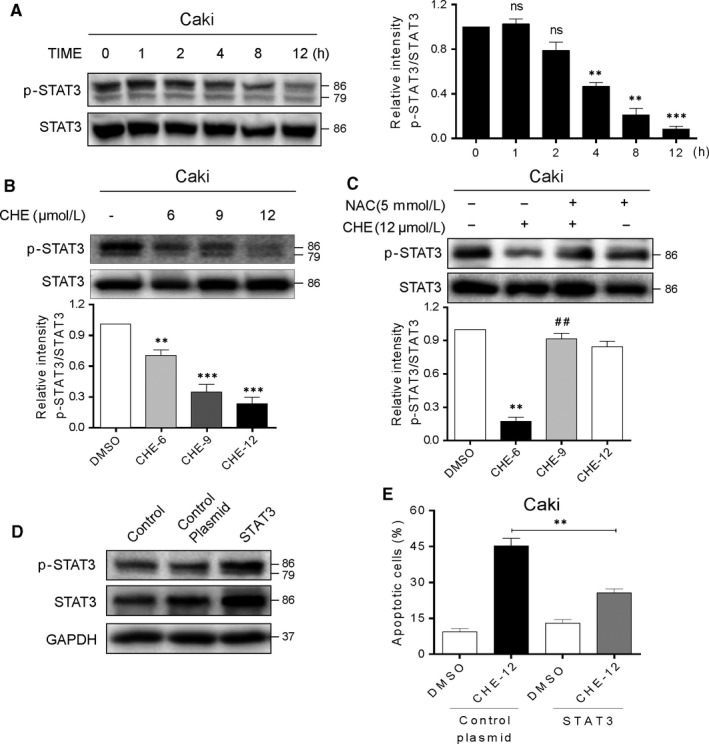
Chelerythrine (CHE) induces apoptosis in renal cancer cells by ROS‐dependent STAT3 pathway. (A) The protein level of p‐STAT3 was examined by Western blot after treatment with CHE (12 μmol/L) for the indicated times in Caki cells. Quantification of data presented in the left panel. (B) Caki cells were treated with CHE (6, 9 or 12 μmol/L) for 10 h, the p‐STAT3 expression was detected by western blot. STAT3 were used as internal control. (C) Caki cells were pretreated with or without 5 mmol/L NAC before exposure to CHE (12 μmol/L) for 10 h, the expression of p‐STAT3 was detected by western blot. STAT3 were used as internal control. (D) Western blotting analysis of stable overexpression of STAT3 protein in Caki cells after STAT3 plasmid transfection [Control = no transfection, control plasmid = control vehicle vector, STAT3 = STAT3 plasmid transfection]. (E) STAT3 overexpressing cells and vector control transfected cells were exposed to CHE and the cell apoptosis was measured by Annexin‐V/PI staining and flow cytometry. All images shown here are representative of three independent experiments with similar results. Data are shown as mean ± SEM (n = 3). **P < 0.01 and ***P < 0.001 compared with the dimethylsulfoxide group; ^##^ *P* < 0.01 compared with the CHE‐12 group

STAT3 plays a key role in cell proliferation through transcriptional activation of pro‐survival genes. In addition, the STAT3 signalling inhibition may promote apoptosis in human cancers. Firstly, we tested the effect of CHE on the expression of cell proliferation markers transcriptionally regulated by STAT3. Our data showed that CHE diminished the constitutive phosphorylation at Y705 residues of STAT3 in Caki cells (Figure 6A,B). Exposure of cells to CHE reduced the p‐STAT3 levels but had no effect on total STAT3 levels. All these effects changed in both a dose‐ and time‐dependent manner (Figure 6A,B). Next, we explored the upstream of STAT3 pathway. Since ER stress pathway was induced by the accumulation of ROS, we conjectured that the productive ROS could inactivate the STAT3 activity. As shown in Figure 6C, pre‐treatment of NAC, the specific ROS inhibitor, for 1 hour significantly reversed the CHE‐induced change in p‐STAT3 level. Then, we confirmed the involvement of STAT3 in CHE‐induced cytotoxic effects through overexpressing STAT3. As shown in Figure 6D, we transfected cells with STAT3 expressing plasmid to increase STAT3 and p‐STAT3 levels in Caki cells. Our results showed that overexpression of STAT3 reduced apoptosis caused by CHE in Caki cells (Figure 6E). All these data demonstrated that the inhibitory activity of CHE in human renal cancer cells was, at least partly, mediated through the inactivation of STAT3.

## 4 DISCUSSION

**Figure 7:**
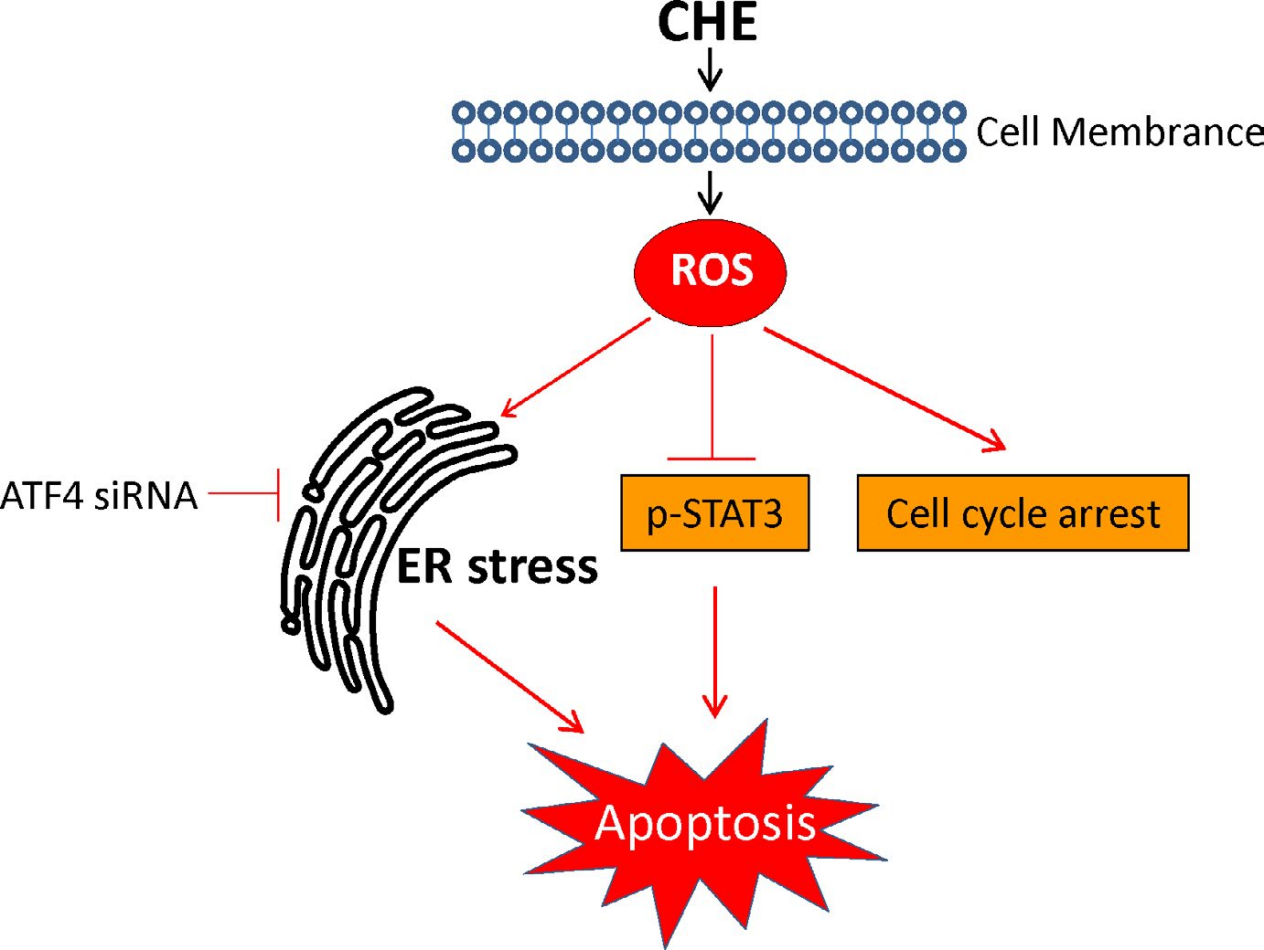
Schematic illustration of the underlying mechanism of CHE's anti‐cancer activity

CHE is considered an anti‐cancer drug but its underlying mechanism has not been well defined yet. In this study, we provided sufficient evidence to demonstrate that CHE inhibited RCC cells growth through by causing cell cycle arrest apoptosis. We also discovered that CHE produced these beneficial inhibitory effects in RCC cells through ROS generation. CHE also induced activation of ER stress and suppression of STAT3 in RCC cells. Knockdown of ATF4 or overexpression of STAT3 both altered the CHE‐induced apoptotic cells. CHE induced apoptosis via ROS‐mediated ER stress and STAT3 pathways in human RCC cells. These salient findings are summarized in Figure 7. Collectively, all of these results suggest that CHE has the potential to be a promising candidate for RCC treatment.

In general, the proliferation of normal cells could be controlled by cell cycle progression, while many cancer cells often lack this regulation.^19^ Our data showed that CHE inhibited the proliferation in RCC cells by causing cell cycle arrest in G2/M phase. The G2/M phase in cell cycle has potential therapeutic effects as various cancer cells always respond effectively to chemotherapy and/or radiation in this cell period.^20^ In addition, the researchers have found that CHE can induce G1 phase arrest of human leukaemia and NSCLC cells in the recent study.^21^, ^22^ This contradiction may be due to the differences of cell types, so the effects of CHE in cell cycle progression should be further studied in more cancer cell lines.

Maintaining moderate levels of intracellular ROS under physiological conditions, for keeping redox balance as well as cell proliferation are extremely important.^23^ ROS is a key regulator of ER stress which generally triggers unfolded protein reaction to protect cancer cells away from cell death.^24^, ^25^ We found that the p‐eIF2a and ATF4 protein levels were increased in a dose‐dependent manner after CHE treatment. Intracellular ROS phosphorylates eIF2a inhibited the overall translation, but selectively promoted the ATF4 translation. Then ATF4 activated the expression of CHOP, a transcription factor. It can up‐regulate various genes involved in cell apoptosis. We next altered ATF4 levels using ATF4 siRNA in cells and assessed the effect of CHE and the results showed that knockdown of ATF4 by siRNA markedly reduced the CHE‐induced apoptosis. It is worth noting that we used five different ROS scavengers (NAC, BHA, Vita‐E, Trolox and CTH) to narrow ROS species. Interestingly, only thiol‐containing antioxidants (NAC) could reverse ROS generation induced by CHE in RCC, while other ROS scavengers did not receive this effect. It showed that lipid ROS inhibitor or lipid peroxide quenchers could not inhibit CHE‐induced ROS, so that the lipid ROS or lipid peroxide may not be involved in CHE treatment. Similarly, only NAC inhibited CHE‐induced cell apoptosis. Thus, ROS species in glutathione and thioredoxin systems may be responsible in CHE actions.

A recent in vitro study on human NSCLC cells, A549 and SK‐MES‐1 cells showed that CHE inhibited cell proliferation when used in combination with erlotinib.^26^ The combination treatment of CHE and erlotinib effectively blocked the EGFR signalling pathway through reducing phosphorylation in downstream targets such as STAT3, ERK1/2 and p38 MAPK. It is worth noting that this suppression of signalling was found in the combination treatment and it is not clear whether CHE along inhibits the pathway. STAT3 (a member of STAT transcription factors) mediates multi aspects of proliferation, immunity, apoptosis and differentiation.^27^, ^28^ Since CHE induced both STAT3 inhibition and ER stress activation, it would be interesting to study the relationship between the two effects. Furthermore, recent studies have found that the increasing ROS level is an important factor leading to apoptosis induced by protein misfolding and ER stress. It has been also reported that ROS inhibits the STAT3 signalling pathway. Thus, we speculate that ROS may be a common upstream mediator of ER stress and STAT3 signalling pathways.^29^, ^30^ The constitutive activation of STAT3 signalling pathway has high frequency detection in many human cancer cell lines or tumours, and including RCC.^31^, ^32^, ^33^

In conclusion, we have identified the anti‐tumour activity of CHE against RCC cells and its potential underlying mechanisms and found that CHE could induce ROS‐dependent cell cycle arrest in G2/M phase and apoptosis. Furthermore, we demonstrate that CHE induced cells apoptosis mainly through activation of ER stress pathway and inhibition of STAT3 phosphorylation. This inhibitory activity is partly reversed by ATF4 knockdown or STAT3 overexpression. Taken together, our findings not only show CHE is a promising candidate for RCC therapy but also indicate that targeting ER stress and STAT3 is a significant strategy for the development of novel anti‐RCC drugs.

## CONFLICT OF INTEREST

All authors declare no conflicts of interest.

## AUTHOR CONTRIBUTIONS

HH and RZ performed the research. JD and XW designed the research study. XH contributed essential reagents or tools. HW analysed the data. DX wrote the manuscript. All authors have read and approved the final manuscript.

## References

[1] Znaor A , Lortet‐Tieulent J , Laversanne M , Jemal A , Bray F . International variations and trends in renal cell carcinoma incidence and mortality. Eur Urol. 2015;67(3):519‐530. 25449206 10.1016/j.eururo.2014.10.002

[2] Jemal A , Siegel R , Ward E , Murray T , Xu J , Thun M . Cancer statistics, 2007. CA Cancer J Clin. 2007;57(1):43‐66. 17237035 10.3322/canjclin.57.1.43

[3] Gupta K , Miller J , Li J , Russell M , Charbonneau C . Epidemiologic and socioeconomic burden of metastatic renal cell carcinoma (mRCC): a literature review. Cancer Treat Rev. 2008;34(3):193‐205. 18313224 10.1016/j.ctrv.2007.12.001

[4] Janzen N , Kim H , Figlin R , Belldegrun A . Surveillance after radical or partial nephrectomy for localized renal cell carcinoma and management of recurrent disease. Urol Clin North Am. 2003;30(4):843‐852. 14680319 10.1016/s0094-0143(03)00056-9

[5] Amato R . Chemotherapy for renal cell carcinoma. Semin Oncol. 2000;27(2):177‐186. 10768596

[6] Li‐Weber M . Targeting apoptosis pathways in cancer by Chinese medicine. Cancer Lett. 2013;332(2):304‐312. 20685036 10.1016/j.canlet.2010.07.015

[7] Gurib‐Fakim A . Medicinal plants: traditions of yesterday and drugs of tomorrow. Mol Aspects Med. 2006;27(1):1‐93. 16105678 10.1016/j.mam.2005.07.008

[8] McChesney J , Venkataraman S , Henri J . Plant natural products: back to the future or into extinction? Phytochemistry. 2007;68(14):2015‐2022. 17574638 10.1016/j.phytochem.2007.04.032

[9] Malíková J , Zdarilová A , Hlobilková A , Ulrichová J . The effect of chelerythrine on cell growth, apoptosis, and cell cycle in human normal and cancer cells in comparison with sanguinarine. Cell Biol Toxicol. 2006;22(6):439‐453. 16964588 10.1007/s10565-006-0109-x

[10] Colombo M , Bosisio E . Pharmacological activities of Chelidonium majus L. (Papaveraceae). Pharmacol Res. 1996;33(2):127‐134. 8870028 10.1006/phrs.1996.0019

[11] Zheng W , Qiu L , Wang R , et al. Selective targeting of PPARγ by the natural product chelerythrine with a unique binding mode and improved antidiabetic potency. Scientific reports. 2015;5:12222. 26183621 10.1038/srep12222 PMC4505335

[12] Tang ZH , Cao WX , Wang ZY , et al. Induction of reactive oxygen species‐stimulated distinctive autophagy by chelerythrine in non‐small cell lung cancer cells. Redox biology. 2017;12:367‐376. 28288416 10.1016/j.redox.2017.03.009 PMC5349618

[13] Yang X , Miao F , Yao Y , et al. In vitro antifungal activity of sanguinarine and chelerythrine derivatives against phytopathogenic fungi. Molecules. 2012;17(11):13026‐13035. 23124471 10.3390/molecules171113026 PMC6268840

[14] Niu X , Mu Q , Li W , Huang H , Yao H , Li H . Protective effects of chelerythrine against lipopolysaccharide‐induced endotoxic shock in mice. Inflammation. 2014;37(6):1968‐1975. 24928629 10.1007/s10753-014-9929-7

[15] Zhang Z , Guo Y , Zhang J , Wei X . Induction of apoptosis by chelerythrine chloride through mitochondrial pathway and Bcl‐2 family proteins in human hepatoma SMMC‐7721 cell. Arch Pharm Res. 2011;34(5):791‐800. 21656365 10.1007/s12272-011-0513-5

[16] Wu S , Yang Y , Li F , et al. Chelerythrine induced cell death through ROS‐dependent ER stress in human prostate cancer cells. OncoTargets and Therapy. 2018;11:2593‐2601. 29780252 10.2147/OTT.S157707 PMC5951218

[17] Lin W , Huang J , Yuan Z , Feng S , Xie Y , Ma W . Protein kinase C inhibitor chelerythrine selectively inhibits proliferation of triple‐negative breast cancer cells. Scientific Reports. 2017;7(1):2022. 28515445 10.1038/s41598-017-02222-0 PMC5435721

[18] Tang J , Farooqi A , Ou‐Yang F , et al. Oxidative stress‐modulating drugs have preferential anticancer effects ‐ involving the regulation of apoptosis, DNA damage, endoplasmic reticulum stress, autophagy, metabolism, and migration. Semin Cancer Biol. 2018. 10.1016/j.semcancer.2018.08.010 30149066

[19] Kamb A , Gruis N , Weaver‐Feldhaus J , et al. A cell cycle regulator potentially involved in genesis of many tumor types. Science. 1994;264(5157):436‐440. 8153634 10.1126/science.8153634

[20] Salazar‐Roa M , Malumbres M . Fueling the cell division cycle. Trends Cell Biol. 2017;27(1):69‐81. 27746095 10.1016/j.tcb.2016.08.009

[21] Vrba J , Dolezel P , Vicar J , Modrianský M , Ulrichová J . Chelerythrine and dihydrochelerythrine induce G1 phase arrest and bimodal cell death in human leukemia HL‐60 cells. Toxicol In Vitro. 2008;22(4):1008‐1017. 18358694 10.1016/j.tiv.2008.02.007

[22] Yang R , Tavares M , Teixeira S , et al. Toward chelerythrine optimization: Analogues designed by molecular simplification exhibit selective growth inhibition in non‐small‐cell lung cancer cells. Bioorg Med Chem. 2016;24(19):4600‐4610. 27561984 10.1016/j.bmc.2016.07.065

[23] Martin K , Barrett J . Reactive oxygen species as double‐edged swords in cellular processes: low‐dose cell signaling versus high‐dose toxicity. Hum Exp Toxicol. 2002;21(2):71‐75. 12102499 10.1191/0960327102ht213oa

[24] Wu W . The signaling mechanism of ROS in tumor progression. Cancer Metastasis Rev. 2006;25(4):695‐705. 17160708 10.1007/s10555-006-9037-8

[25] Boonstra J , Post J . Molecular events associated with reactive oxygen species and cell cycle progression in mammalian cells. Gene. 2004;337:1‐13. 15276197 10.1016/j.gene.2004.04.032

[26] He M , Yang Z , Zhang L , Song C , Li Y , Zhang X . Additive effects of cherlerythrine chloride combination with erlotinib in human non‐small cell lung cancer cells. PloS One. 2017;12(4):e0175466. 28399187 10.1371/journal.pone.0175466 PMC5388488

[27] Darnell J , Kerr I , Stark G . Jak‐STAT pathways and transcriptional activation in response to IFNs and other extracellular signaling proteins. Science. 1994;264(5164):1415‐1421. 8197455 10.1126/science.8197455

[28] Yu H , Pardoll D , Jove R . STATs in cancer inflammation and immunity: a leading role for STAT3. Nat Rev Cancer. 2009;9(11):798‐809. 19851315 10.1038/nrc2734 PMC4856025

[29] Zhang J , Ahn K , Kim C , et al. Nimbolide‐induced oxidative stress abrogates STAT3 signaling cascade and inhibits tumor growth in transgenic adenocarcinoma of mouse prostate model. Antioxid Redox Signal. 2016;24(11):575‐589. 26649526 10.1089/ars.2015.6418

[30] Chen X , Dai X , Zou P , et al. Curcuminoid EF24 enhances the anti‐tumour activity of Akt inhibitor MK‐2206 through ROS‐mediated endoplasmic reticulum stress and mitochondrial dysfunction in gastric cancer. Br J Pharmacol. 2017;174(10):1131‐1146. 28255993 10.1111/bph.13765 PMC5406301

[31] Banerjee K , Resat H . Constitutive activation of STAT3 in breast cancer cells: a review. Int J Cancer. 2016;138(11):2570‐2578. 26559373 10.1002/ijc.29923 PMC4801660

[32] Haura E , Zheng Z , Song L , Cantor A , Bepler G . Activated epidermal growth factor receptor‐Stat‐3 signaling promotes tumor survival in vivo in non‐small cell lung cancer. Clin Cancer Res. 2005;11(23):8288‐8294. 16322287 10.1158/1078-0432.CCR-05-0827

[33] Guo Y , Xu F , Lu T , Duan Z , Zhang Z . Interleukin‐6 signaling pathway in targeted therapy for cancer. Cancer Treat Rev. 2012;38(7):904‐910. 22651903 10.1016/j.ctrv.2012.04.007

