# Supplementary figures and images for: Chelerythrine induces apoptosis via ROS‐mediated endoplasmic reticulum stress and STAT3 pathways in human renal cell carcinoma
Source: J Cell Mol Med. 2019 Sep 30;24(1):50–60. doi: 10.1111/jcmm.14295 (PMC6933352; doi:10.1111/jcmm.14295)

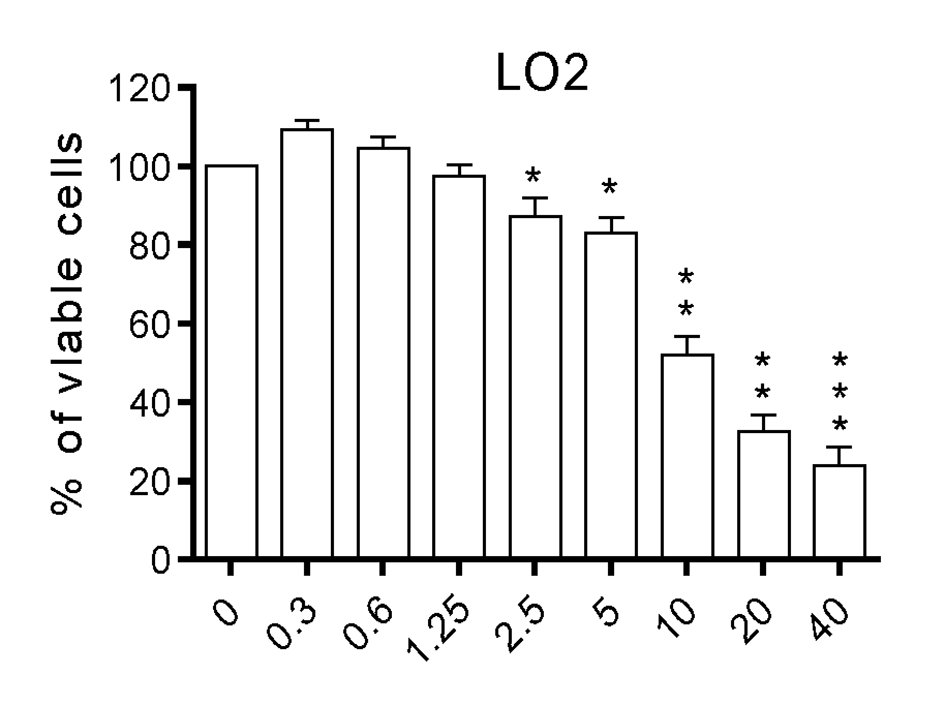

Supplement: Supplementary file 1 [file JCMM-24-50-s001.tif]

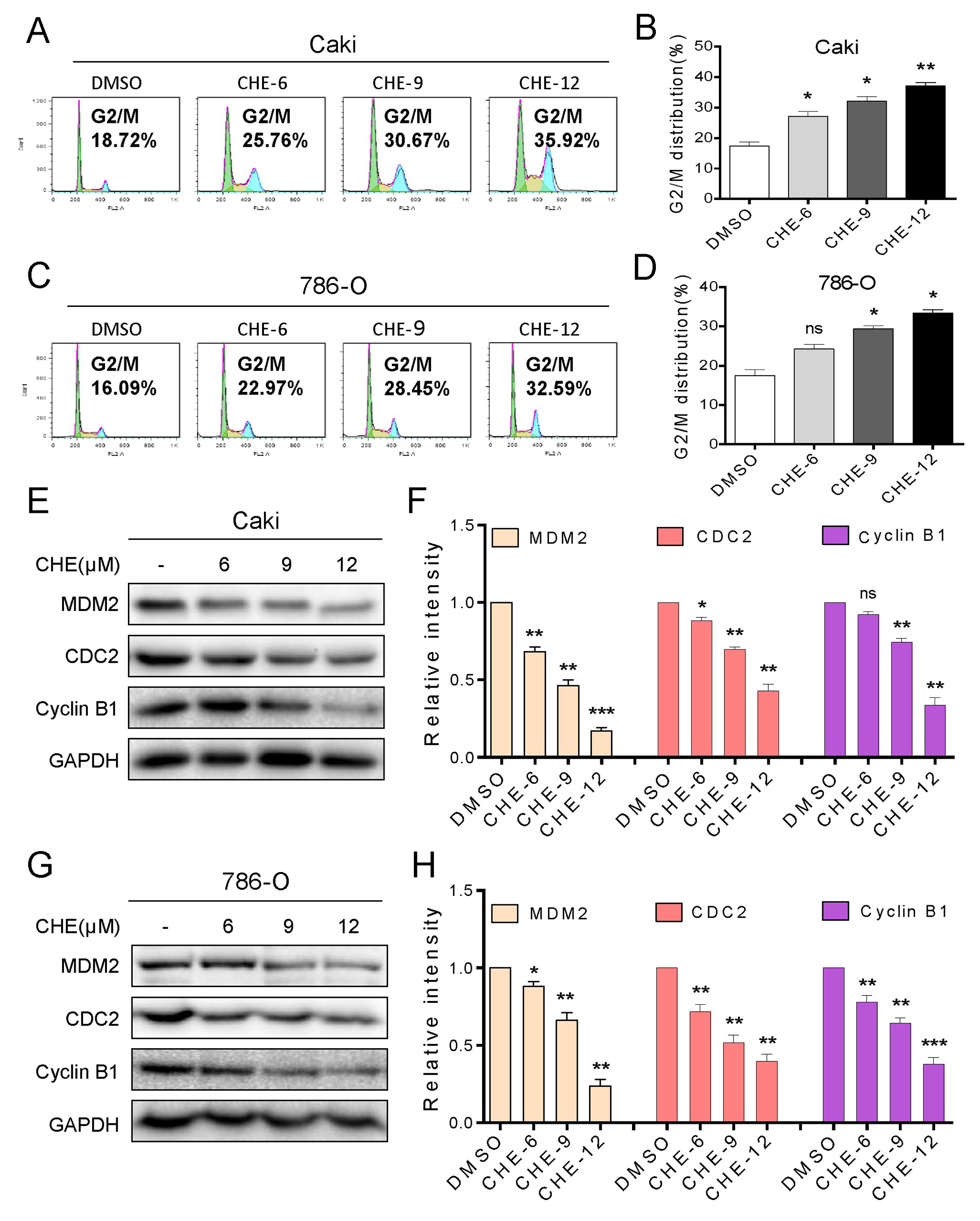

Supplement: Supplementary file 2 [file JCMM-24-50-s002.tif]

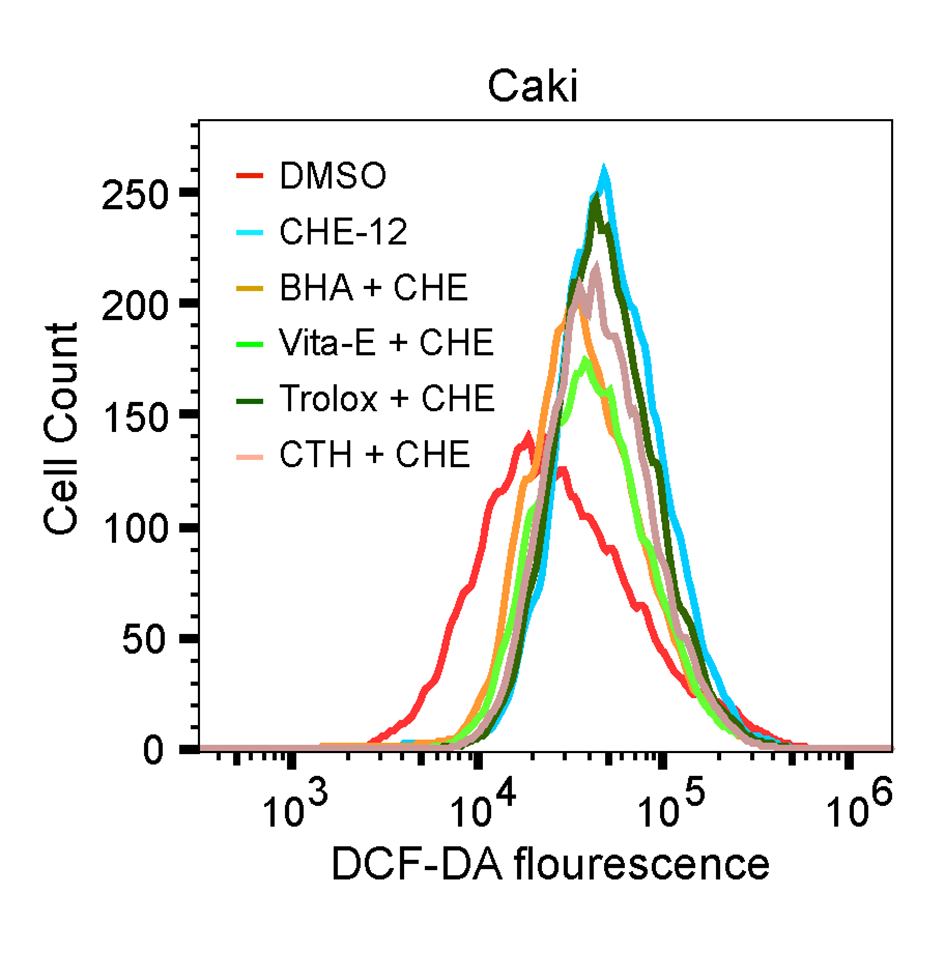

Supplement: Supplementary file 3 [file JCMM-24-50-s003.tif]

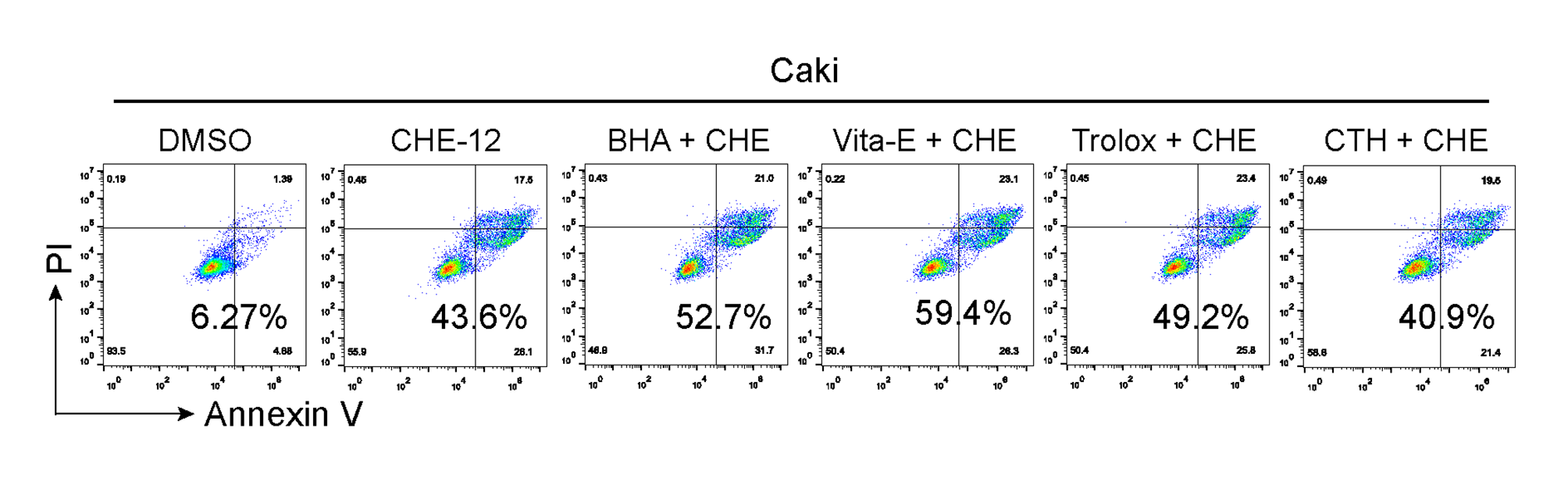

Supplement: Supplementary file 4 [file JCMM-24-50-s004.tif]

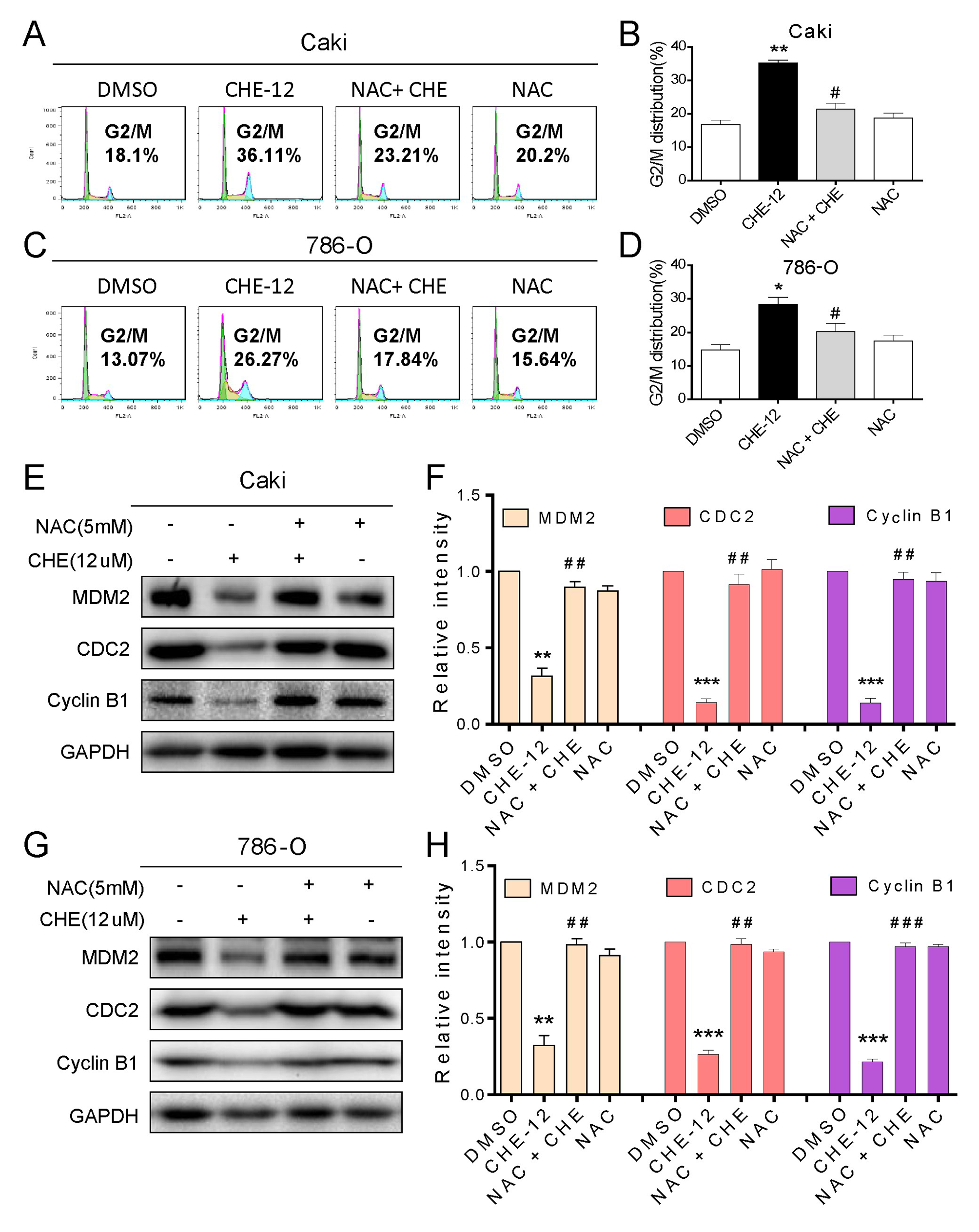

Supplement: Supplementary file 5 [file JCMM-24-50-s005.tif]
